# Unequal representation of genetic variation across ancestry groups creates healthcare inequality in the application of precision medicine

**DOI:** 10.1186/s13059-016-1016-y

**Published:** 2016-07-14

**Authors:** Slavé Petrovski, David B. Goldstein

**Affiliations:** Institute for Genomic Medicine, Columbia University, New York, New York USA; Department of Medicine, The University of Melbourne, Austin Health and Royal Melbourne Hospital, Melbourne, Victoria Australia

**Keywords:** Clinical diagnostics, Precision medicine, Disease-associated genes, Healthcare inequality, Genetic ancestry, Genetic variation, Geographic ancestry, Next generation sequencing, Rare variants, Sequence interpretation

## Abstract

An important application of modern genomics is diagnosing genetic disorders. We use the largest publicly available exome sequence database to show that this key clinical service can currently be performed much more effectively in individuals of European genetic ancestry.

**Electronic supplementary material:**

The online version of this article (doi:10.1186/s13059-016-1016-y) contains supplementary material, which is available to authorized users.

## Background

It has long been argued that the concentration of large scale genomic data generation on individuals of European ancestry can contribute to healthcare inequalities [1, 2]. Currently, in the search for a genetic diagnosis, much of the effort in the diagnostic sequencing paradigm is focused on candidate variants among known disease-associated genes that are either absent or sufficiently rare in available control reference cohorts, each of which is considered carefully as a possible explanation for the relevant presentation. Need and Goldstein specifically argued in 2009 that our ability to effectively filter variants to identify pathogenic ones as sequencing becomes clinically routine would be very different amongst different ancestry groups unless our knowledge of genetic variation is made more equal across ancestry groups [1]. Unfortunately, now with clinical sequencing becoming routine this fear has been clearly realized. The common experience is that when this clinical service is done today in patients of European ancestry, the number of candidate variants is significantly less than in other geographic ancestry groups.

When searching for genetic aberrations responsible for Mendelian disorders, the expectation that pathogenic genotypes will be under strong negative selection instructs us to focus on genotypes at low or unobserved frequencies in the general population [3–5]. As population reference cohorts increase in size we capture lower allele frequencies with improved resolution [6]. The recently released Exome Aggregation Consortium (ExAC) dataset [7, 8], which contains aggregated exome sequence data from 60,252 individuals with an assigned geographic ancestry, aids in identifying allelic frequencies at an approximately sixfold lower resolution than what was available from the combination of two pre-existing datasets, the Exome Sequencing Project (ESP) and the 1000 Genomes Project. Approximately 60.9 % of the samples in this ExAC reference cohort are of European ancestry, compared with 13.7 % of South Asian ancestry, 9.6 % of Latino ethnicity, 8.6 % of African (African American) ancestry, and 7.2 % of East Asian ancestry.

**Fig. 1:**
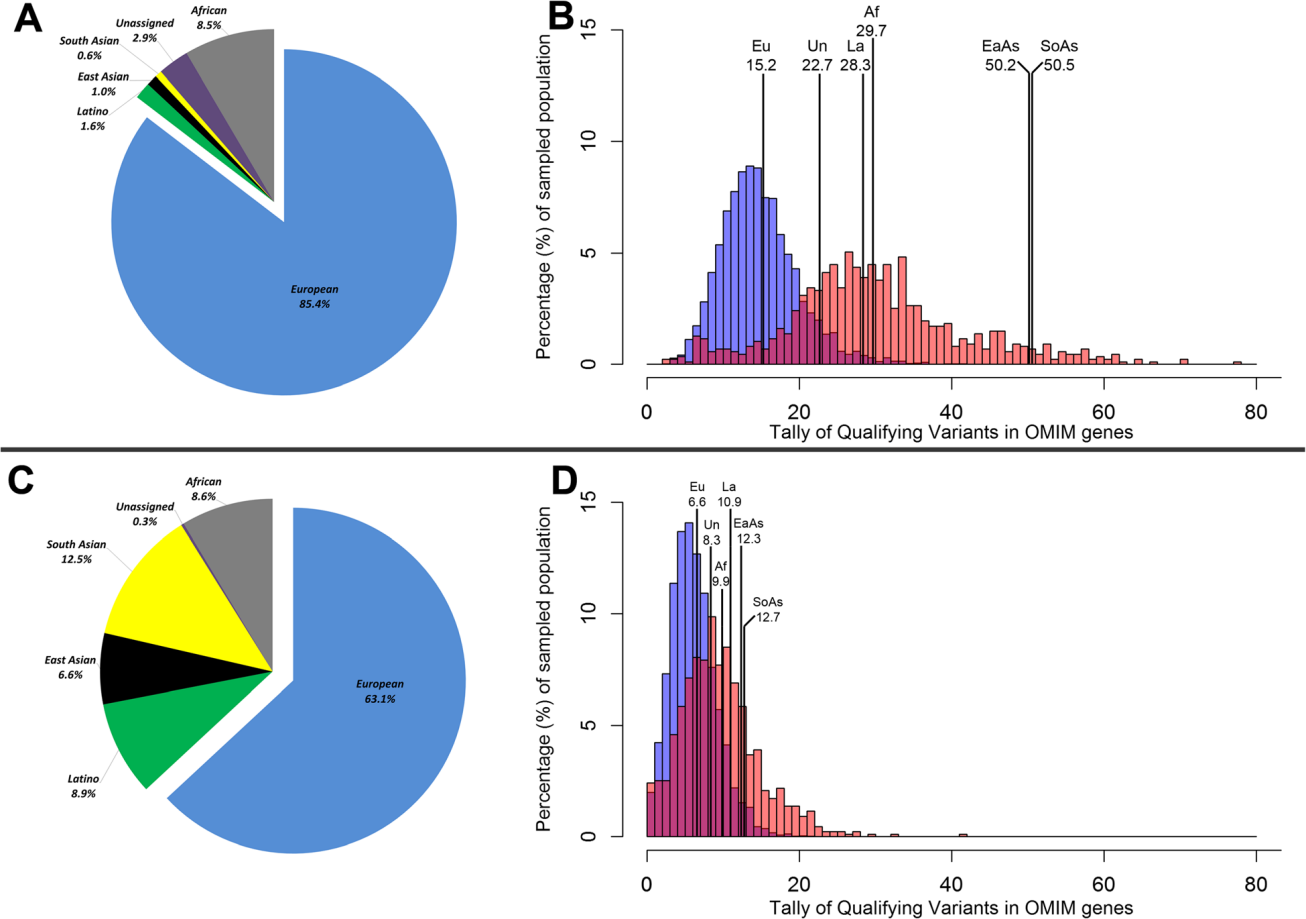
**a** Percentage representation of the 5965 IGM reference cohort across six geographic ancestry groupings. **b** A semi-transparent overlaid histogram representing the tally of candidate variants between IGM’s 5094 European (*Eu*) individuals (*blue*) and the collection of non-European individuals (*red*) (Mann–Whitney U test *p* < 1 × 10^−320^). The non-European distribution reflects individuals with a: Latino ethnicity (*La*), East Asian (*EaAs*), South Asian (*SoAs*), primarily African (*Af*), and unassigned (*Un*) ancestry. Estimates indicate the mean number of singleton non-synonymous variants among OMIM disease-associated genes. Singleton variants are identified based on a reference cohort of 5965 IGM sequenced samples. **c** Percentage representation of the combined 66,217 IGM and ExAC reference cohorts across six geographic ancestry / ethnic groupings. **d** Similar to **b** but singleton variants were identified based on the absence among the combined IGM and ExAC reference cohorts accumulating to 66,217 samples

Here, we evaluate the consequence of geographic ancestry on the effectiveness of interpreting a genome among a collection of 5965 individuals sequenced for various studies at the Institute for Genomic Medicine (IGM). We use a principal component (PC) approach [9] to assign samples into geographic ancestry groups (Additional file 1). Our cohort comprises 5094 (85.4 %) individuals of European genetic ancestry, 505 (8.5 %) of primarily African ancestry, 93 (1.6 %) of Latino ethnicity, 61 (1 %) of East Asian ancestry, and 38 (0.6 %) of South Asian ancestry; 174 (2.9 %) samples were allocated to an “unassigned” ancestry group (Fig. 1a).

## Geographic ancestry, rare variants, and disease-associated genes

We previously described “narrative potential” [10] as the opportunity to construct variant-disease narratives given that every genome will contain rare variants predicted to be damaging by in silico tools. To illustrate the value of ancestry matched controls, we generated rare variant distributions for the different ancestry groups. The distributions reflect the number of rare non-synonymous variants found among the 3393 current disease-associated genes from the Online Mendelian Inheritance in Man (OMIM) database.

The first assessment (Fig. 1b) compares the European (blue) and non-European (red) distribution for the number of singleton non-synonymous variants each sample has among OMIM disease-associated genes (Additional file 1). The minor allele frequency (MAF) is based on the internal database of 5965 IGM samples. Due to the reduced access to ethnically matched controls, when comparing the distribution between the European and non-European ancestries, we find longer candidate lists among non-Europeans (Mann–Whitney U test *p* < 1 × 10^−320^).

After further removing variants reported in the ExAC reference cohort (Fig. 1c) [7, 8], individuals with European ancestry have, on average, 6.6 candidate singleton non-synonymous variants that overlap OMIM disease-associated genes. In comparison, we see 9.9 candidate variants in individuals with primarily African, 10.9 in Latino ethnicity, 12.3 in East Asian, 12.7 in South Asian, and 8.3 in the unassigned ancestry group (Table 1). While this illustrates that growing and diverse datasets are a critical step towards harmonizing the distribution of candidate variants, it is evident that the problem is not yet solved (Fig. 1d; Mann–Whitney U test *p* = 5 × 10^−91^). As a simple illustration, randomly selecting a European representative finds six candidate OMIM gene variants, one within a dominant gene. In comparison, randomly selecting a South Asian representative results in 13 candidate OMIM gene variants, eight occurring in dominant genes (Additional file 1). This is a challenge currently faced by research, clinical, and diagnostic sequencing labs. While the numerical difference sounds small, when you consider that you may need to act on the basis of the patients’ genetic diagnosis, every additional candidate has a true implication on interpretation.

**Table 1:** Group summaries for the number of singleton non-synonymous candidate variants in OMIM disease-associated genes among IGM’s 5965 samples

Geographic ancestry / ethnic group	Number of individuals	Number of singletons using internal reference cohort (n = 5,965)			Number of singletons using internal and ExAC reference cohorts (n = 66,217)		
		Median	Mean	SD	Median	Mean	SD
European	5,094	15	15.2	5.0	6	6.6	3.0
African (African American)	505	29	29.7	8.3	9	9.9	4.8
Latino ethnicity	93	28	28.3	6.0	10	10.9	5.5
East Asian	61	51	50.2	9.1	12	12.3	4.2
South Asian	38	49.5	50.5	7.4	12	12.7	3.8
Unassigned	174	24	22.7	11.9	8	8.3	5.8

Data reflect using only the internal reference cohort and then subsequently supplementing the IGM internal reference cohort with variant information from the ExAC reference cohort of 60,252 controls of convenience

*SD* standard deviation

## Conclusions

These analyses illustrate how unequal representation of genetic variation can negatively affect present genomic interpretation in individuals of non-European ancestry. While the results are unsurprising given our understanding of population genetics, there are still important lessons. Firstly, these data show that it is instructive to assess the allele frequencies of non-European cases in their matched ancestry group(s). Secondly, increasing diversity of geographic ancestry and sample size among sequenced reference cohorts greatly ameliorates the problem (Fig. 1).

Given that sample sizes are about to explode with the US national initiative and other large-scale international sequencing studies, it is vital that we ensure the most equitable distribution of the generation of genomic data possible. Enriching our knowledge of genetic variation in different ancestry groups remains the most effective solution to this problem. With initiatives like the recently announced Precision Medicine Initiative (PMI) Cohort Program, this must be recognized as a high priority for the field as we move towards an era where precision medicine is a reality. If not, genomics could further contribute to healthcare inequalities.

## Abbreviations

ExAC, Exome Aggregation Consortium; IGM, Institute for Genomic Medicine; OMIM, Online Mendelian Inheritance in Man; PC, principal component

## Additional file

Additional file 1: Supplementary methods that describe the variant calling quality control parameters adopted and the principal component ancestry predictions. (PDF 390 kb)
