# Supplementary material for: Unequal representation of genetic variation across ancestry groups creates healthcare inequality in the application of precision medicine
Source: Genome Biol. 2016 Jul 14;17:157. doi: 10.1186/s13059-016-1016-y (PMC4944427; doi:10.1186/s13059-016-1016-y)
Supplement: Additional file 1: — Supplementary methods that describe the variant calling quality control parameters adopted and the principal component ancestry predictions. (PDF 390 kb) [file 13059_2016_1016_MOESM1_ESM.pdf]

Supplementary Data:

Principal component (PC) used to classify ancestry group

Classifying sequenced samples into a major geographic ancestry group was achieved by generating principal components (PCs) based on 3,325 well-covered exonic polymorphic markers and then taking predictions from a multinomial logistic regression model that uses the first five PC axes as the independent variables and is trained on 4,811 individuals for whom we have existing human evaluated PC-based genetic ancestry. Each sample is assigned a probability estimate for each of six ancestry groups (European, African American, Latino, East Asian, South Asian and Middle Eastern), with the sum of probabilities equalling one. Each sample is allocated to the ancestry group for which it achieved a Pr(Group)≥0.80. Samples that did not achieve a Pr(Group)≥0.80 for a single ancestry group were allocated to a seventh group, referred to as the "unassigned" group. To match our cohort with the ExAC reference cohort, we do not include the small set of samples that were predicted to belong to the Middle Eastern genetic ancestry group. To assess concordance, we compared PC-based ancestry predictions with self-declared geographic ancestry for 5,090 samples and observed 95.8% accuracy. Greater than half of the discordant samples are individuals who were "unassigned" by the PCs, which includes many admixed individuals. As a result, 5,965 samples were assigned to one of the six genetic ancestry groups. These 5,965 samples do not represent multiple individuals from known families, nor any two individuals found to have cryptic relatedness closer than 2nd degree relatives (pairwise identity-by-descent of 0.25). The collection of 5,965 IGM samples also has an increased European representation when compared to the 2000 US population census estimates that approximated 71% of the US population reporting a European ancestry (http://www.census.gov/population/ancestry/data/). In addition to a focus on European populations in research study design there are also cultural

reasons why individuals from some communities are more hesitant to participate in research and in public sharing of de-identified variation data.

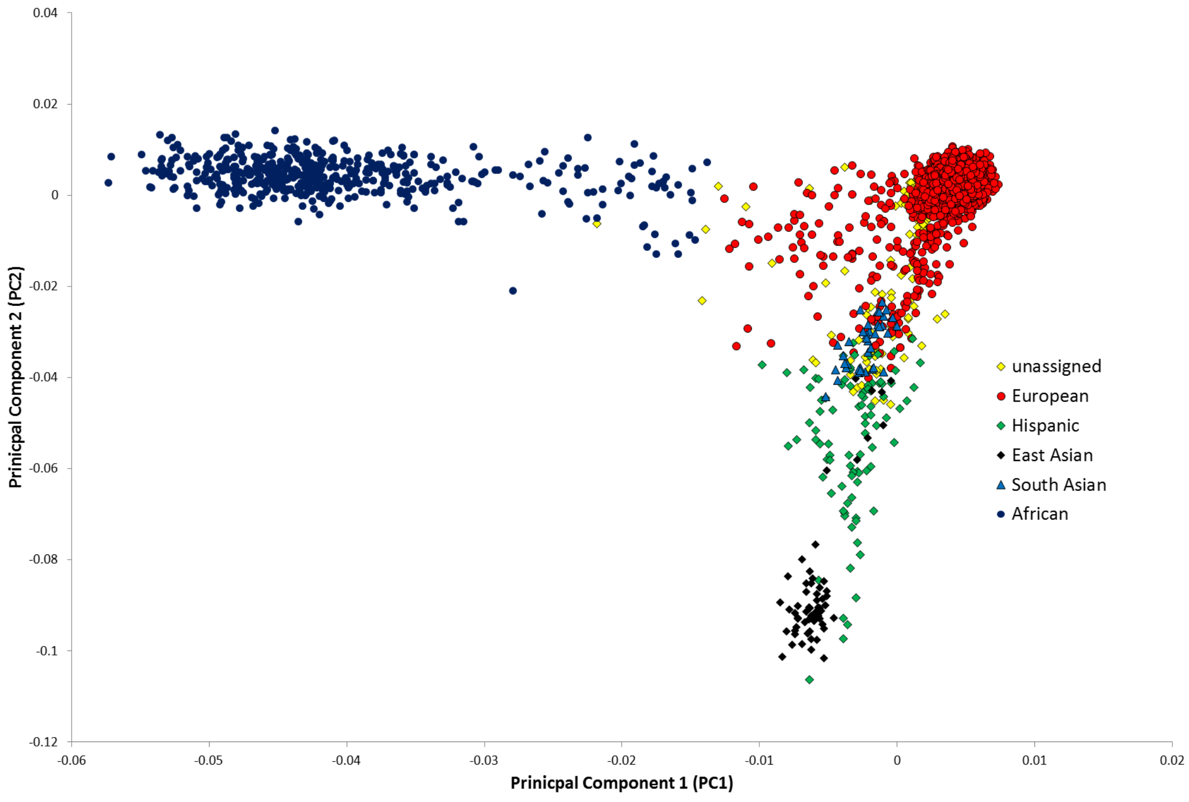

***Figure S1** – Position of the 5,965 CHGV sequenced samples across the first two principal component ancestry axes.*

Variant calling

The cohort of 5,965 samples was sequenced by the Institute for Genomic Medicine (IGM), Columbia University; previously the Center for Human Genome Variation, Duke University. Samples were either exome sequenced using the Agilent All Exon (50MB or 65MB) or the Nimblegen SeqCap EZ V2.0 or 3.0 Exome Enrichment kit or whole-genome sequenced using Illumina HiSeq 2000 or 2500 sequencers according to standard protocols. The Illumina lane-level fastq files were aligned to the Human Reference Genome (NCBI Build 37) using the Burrows-Wheeler Alignment Tool (BWA). We used Picard (http://picard.sourceforge.net) to remove duplicate reads and process lane-level SAM files, resulting in a sample-level BAM

file that was used for variant calling. To call variants, we used GATK best practice protocol, recalibrated base quality scores, and realigned around indels.

Only non-synonymous single nucleotide substitution variants residing within the consensus coding sequence (CCDS) release 14 sequence were considered. Variants were excluded if they were among a predefined in-house list of sequencing artifacts. SNVs were required to have: i) at least 10-fold coverage in index case and at least 10-fold average coverage at site within the ExAC database, ii) a quality score (QUAL) of at least 50, iii) a genotype quality (GQ) score of at least 20 with an alternative allele frequency between 30-70% of reads for heterozygous genotypes, iv) a quality by depth (QD) score of at least 5, v) a mapping quality (MQ) score of at least 40, vi) a read position rank sum (RPRS) score greater than -8 and vii) mapping quality rank sum (MQRS) score greater than -12. Variants were further screened according to VQSR tranche calculated using the known SNV sites from HapMap v3.3, dbSNP, and the Omni chip array from the 1000 Genomes Project. To "PASS," variants were required to achieve a tranche less than 99.9% for SNVs in genomes and less than 99% for SNVs in exomes.

Comparing a European and non-European sample

Randomly selecting a European ancestry representative we find six candidate variants in OMIM genes: *ASPM, BBS9, EPAS1, HMGCR, MTHFR* and *WDR35*; where only *EPAS1* is linked to disease via a dominant genetic model. In comparison, when we randomly selected a South Asian ancestry representative we found 13 candidate variants in OMIM disease-associated genes: *COL9A3, DIS3L2, EYA1, IL17F, KANK1, NLRP12, OLR1, SCP2, TG, TTN* x 3, and *WDR62*; where eight of these variants occur in genes linked to disease through a dominant genetic model.
